# Psychometric validation of the Female Sexual Distress Scale-Desire/Arousal/Orgasm

**DOI:** 10.1186/s41687-021-00359-1

**Published:** 2021-09-24

**Authors:** Leonard R. Derogatis, Dennis A. Revicki, Raymond C. Rosen, Robert Jordan, Johna Lucas, Carl Spana

**Affiliations:** 1 Maryland Center for Sexual Health, 1228 Wine Spring Lane, Towson, MD 21204 USA; 2 Evidera, Bethesda, MD USA; 3 HealthCore/New England Research Institutes, Watertown, MA USA; 4 Palatin Technologies, Inc., Cranbury, NJ USA

**Keywords:** Female sexual dysfunction, HSDD, FSAD, Bremelanotide, FSDS-DAO, Psychometric characteristics, Patient-reported outcomes, Instrument validation

## Abstract

**Background:**

For the treatment of female sexual dysfunction, the most relevant outcome measures are patient-reported treatment effects and changes in symptoms, underscoring the need for reliable, validated patient-reported outcome (PRO) instruments. The aim of this study was to evaluate the psychometric characteristics (validity and reliability) of the Female Sexual Distress Scale-Desire/Arousal/Orgasm (FSDS-DAO) PRO measure, which was adapted from the validated FSDS-Revised (FSDS-R) questionnaire and added 2 questions involving arousal and orgasm.

**Methods:**

Psychometric analyses were based on the data from a multicenter phase 2b dose-finding study that compared the safety and efficacy of bremelanotide versus placebo and were conducted in the evaluable modified intent-to-treat population (N = 325) from that study. Psychometric evaluation of the new items in the FSDS-DAO included confirmatory factor analyses, tests of internal consistency and test–retest reliability, examinations of convergent and discriminant validity, and determination of responsiveness. The validity of the FSDS-DAO was evaluated based on previously developed instruments, including the Female Sexual Function Index (FSFI), General Assessment Questionnaire (GAQ), Women’s Inventory of Treatment Satisfaction (WITS-9), and Female Sexual Encounter Profile-Revised (FSEP-R).

**Results:**

Confirmatory factor analyses demonstrated that the FSDS-DAO items fit very well (Bentler’s comparative fit index of 0.929). Cronbach’s α for the FSDS-DAO total score was ≥ 0.91 at Visits 1, 2, 5, and 12, demonstrating adequate internal consistency reliability. Test–retest reliability was acceptable with an intra-class coefficient of 0.61 and a Spearman’s correlation coefficient score of 0.62 between Visits 1 and 2 (4 weeks). Acceptable construct validity was demonstrated by significant correlations with related PRO scales in the expected directions and magnitude. For example, participants reporting the worst levels of sexual function on the FSFI also showed the worst FSDS-DAO scores at Visits 5 and 12. The FSDS-DAO total score was responsive to change.

**Conclusions:**

Evidence supports the validity and reliability of the FSDS-DAO for assessing sexually related distress in women with female sexual arousal disorder and/or hypoactive sexual desire disorder; the addition of the arousal and orgasm items did not impact the validity and reliability of the measure.

*Clinical Trial Registration* ClinicalTrials.gov NCT01382719.

**Supplementary Information:**

The online version contains supplementary material available at 10.1186/s41687-021-00359-1.

## Background

Female sexual dysfunction (FSD) comprises a group of common conditions with physiological, psychological, and social components [1]. The most prevalent sexual dysfunction among women is hypoactive sexual desire disorder (HSDD), which is defined as persistent or recurrent diminished or lack of desire for sexual activity accompanied by personal distress, not due to medications or existing medical or relationship issues [2, 3]. The presence of distress experienced by women with HSDD has important implications for diagnosis and treatment [4, 5].

The Female Sexual Distress Scale (FSDS) [6], a 12-item patient-reported outcome (PRO) instrument, was developed to measure sexually related personal distress in women. Both the original 12-item version and the 13-item FSDS-Revised (FSDS-R) [7] version have been shown to have a high degree of internal consistency, test–retest reliability, and discriminative validity to distinguish between sexual function and dysfunction among women. The original FSDS was psychometrically evaluated in 2002 [6]. In 2008, the FSDS-R was created following the addition of Question 13 (bothered by low sexual desire) and was psychometrically evaluated primarily in postmenopausal women [7]. Moderate positive correlations with other conceptually related nonsexual measures of distress have also been noted. In response to recommendations from key FSD opinion leaders and the US Food and Drug Administration (FDA), the FSDS-R was recently modified by the addition of 2 items. This newest version, named the Female Sexual Distress Scale-Desire/Arousal/Orgasm (FSDS-DAO), was also adapted to be completed electronically using a handheld device.

The objective of the current analysis was to evaluate the psychometric characteristics (reliability and validity) of the FSDS-DAO using data from a large (N = 327), multicenter, placebo-controlled, phase 2b, dose-finding study of bremelanotide (PT-141), an investigational, novel cyclic 7-amino acid melanocortin receptor agonist with a high affinity for the type-4 receptor [8] that is currently being evaluated for the treatment of HSDD (with or without decreased arousal) in premenopausal women (ClinicalTrials.gov Identifier: NCT01382719) [9].

## Methods

### Study participants

Details of the phase 2b study have been previously published [9]. Briefly, participants in the study were premenopausal (according to the Stages of Reproductive Aging Workshop [STRAW] criteria), nonpregnant women ≥ 21 years old with HSDD, female sexual arousal disorder (FSAD), or a combination of these disorders for ≥ 6 months prior to the start of the study. Participants were required to have been diagnosed by a qualified clinician using a diagnostic interview and to have a total score > 18 on the FSDS-DAO and a total score < 26.5 on the Female Sexual Function Index (FSFI) [10]. Participants were also required to have had previous sexual functionality for at least 2 years, be currently in a monogamous relationship of ≥ 6 months’ duration, and be willing to be sexually active with this partner ≥ 1 time/month during the study. Exclusion criteria included women who presented with unstable or uncontrolled medical conditions, had a history of unresolved sexual trauma or abuse, had been treated for depression or psychosis within the preceding 6 months, had used antidepressants or antipsychotics within the preceding 3 months, or were undergoing current psychotherapy for FSD. Also excluded were women with lifelong anorgasmia, vaginismus, sexual pain disorder, sexual aversion disorder, or persistent sexual arousal disorder. For inclusion in this analysis, data were derived from participants who had FSDS-DAO scores at baseline and at 1 or more follow-up visits. These 325 subjects comprised the evaluable modified intent-to-treat (MITT) population.

### Study design

This multicenter, randomized, placebo-controlled, dose-finding study was conducted at 68 sites in the United States and Canada. All participants underwent a 4-week, no-treatment screening/qualification period, followed by a 4-week, single-blind self-dosing (placebo-only) period to establish baseline and were then randomized to self-administer placebo or 3 different doses of bremelanotide (0.75, 1.25, or 1.75 mg) as desired over 12 weeks [9]. In the phase 2b study, the primary efficacy endpoint was the change from baseline to the end of the study in the number of satisfying sexual encounters (SSEs) as assessed by the Female Sexual Encounter Profile-Revised questionnaire (FSEP-R Q10) [9]. Other PRO measures were the Female Sexual Distress Scale-Desire/Arousal/Orgasm (FSDS-DAO), Female Sexual Function Index (FSFI), Sexual Interest and Desire Inventory (SIDI-F), General Assessment Questionnaire (GAQ), and Women’s Inventory of Treatment Satisfaction (WITS-9).

PRO outcomes were assessed at various time points throughout the trial to observe changes over time, including baseline, early in the trial, and at the trial endpoint. Time points varied as not all PRO instruments were administered at each time point: FSEP-R was completed after each sexual encounter, while other PRO outcomes were assessed at Weeks 0 and 4 (Visits 1 and 2), with the exception of GAQ and WITS-9, and at Weeks 10, 16, 20, and 23 (Visits 5, 10, 11, and 12, respectively). All PRO instruments were completed by participants using an electronic handheld device (eDiary). In addition, the SIDI-F was also completed via interview by clinical research staff. In this analysis, data from Weeks 0, 4, 10, and 23 were used for psychometric evaluation.

### PRO measures

#### Female Sexual Distress Scale-Desire/Arousal/Orgasm (FSDS-DAO)

The 15-item FSDS-DAO retains the 13 items from the Likert-type FSDS-R scale, which has evidence supporting reliability and validity [6, 7]. The FSDS-DAO includes 2 new items that ask women to rate their level of distress related to arousal and orgasm. As with previous versions of the FSDS, participant responses to “How often did you feel concerned with difficulties with sexual arousal?” and “How often did you feel frustrated by problems with orgasm?” are provided using a polytomous response scale ranging from 0 (never) to 4 (always). Subjects who met eligibility criteria completed the FSDS-DAO with a 30-day recall at baseline and at Visits 2, 5, 10, 11, and 12. The total score is calculated as the sum of the responses and ranges from 0 to 60, with higher scores indicating a greater level of distress. The total score on the FSDS-R can range from 0 to 52 [11]. For the purposes of this analysis, we present data on the FSDS-DAO for Visit 2 (baseline), Visit 5, and Visit 12 because of the 30-day recall period. The windows between Visits 10 and 11 and Visits 11 and 12 are only 28 and 21 days apart, respectively. Thus, Visits 10 and 11 data were not included in the analyses to reduce overlap in the assessments.

Psychometric evaluation of the FSDS-DAO was undertaken against the following PRO instruments described below. In addition, the analysis was repeated using the FSDS-R, which does not include the arousal and orgasm items in order to provide a comparison between it and the FSDS-DAO.

#### Female Sexual Function Index (FSFI)

The FSFI is a 19-item measure of female sexual function consisting of 6 domains: desire, arousal, lubrication, orgasm, satisfaction, and pain [10, 12]. Scores for the arousal, lubrication, orgasm, and pain domains range from 0 to 6 using Likert-type scales. Scores for desire range from 1.2 to 6.0, and those for satisfaction range from 0.8 to 6.0. The total score is the sum of the domain scores and ranges from 2 to 36, and the recall period is the past 4 weeks. Higher scores indicate a better level of sexual function.

#### Female Sexual Encounter Profile-Revised (FSEP-R)

The FSEP-R is a 10-item instrument that is designed to assess sexual encounters, including initiation, level of desire, satisfaction with arousal, lubrication, arousal, ability to achieve orgasm, and satisfaction with the sexual encounter [13]. Participants completed the FSEP-R within 24 h of a sexual encounter. A “sexual encounter” is defined as any act involving sexual contact with genitalia and/or oral mucosa, and includes intercourse, oral sex, and masturbation by self or a partner. Q10 reads “Did you consider this sexual encounter satisfactory for you?” and answers were yes or no.

#### General Assessment Questionnaire (GAQ)

The GAQ consists of 4 items: satisfaction with arousal, desire, degree of benefit while on study drug, and impact of taking study drug on relationship with partner. Responses are selected on a 7-point numeric rating scale from 1 (very much worse) to 7 (very much better). A score ≥ 5 indicates benefit.

#### Women’s Inventory of Treatment Satisfaction (WITS-9)

The validated WITS-9 questionnaire assesses satisfaction with treatment and sexual relations over the past 4 weeks [14]. Participants answer the 9 items on a 7-point numeric rating scale from − 3 (very unsatisfied or very likely not to continue) to 3 (very satisfied or very likely to continue). The total score is calculated as the average of the scores from the 9 questions and ranges from − 3.0 to 3.0. A higher score on the WITS-9 indicates a higher level of satisfaction with treatment.

### Statistical analysis

Specific statistical tests are described above for each endpoint. All analyses were performed using SAS version 9.2 or later. All statistical tests were conducted with conservative decision-making criteria established a priori according to published guidance [15]. Missing data were considered missing, and no data imputations were performed. All statistical tests were 2-tailed and were conducted with type I error probability fixed at 0.05. For continuous variables, the mean and standard deviation were described; for categorical variables, the percent distribution by category was described.

### FSDS-DAO psychometric evaluation

#### Instrument descriptive characteristics

Individual item performance and frequency of responses on the FSDS-DAO and FSDS-R items and total scores, including rates of missing data, were examined at Visit 1 (Week 0), Visit 2 (Week 4), Visit 5 (Week 10), Visit 11 (Week 19), and Visit 12 (Week 23). Individual item performance and frequency of responses on the FSEP-R item scores, including rates of missing data, were examined at Visit 2 (Week 4), Visit 5 (Week 10), Visit 11 (Week 19), and Visit 12 (Week 23). Distributional characteristics of the FSFI were examined at Visit 1 (Week 0), Visit 2 (Week 4), Visit 5 (Week 10), Visit 11 (Week 19), and Visit 12 (Week 23). The GAQ and WITS-9 were examined at Visit 5 (Week 10), Visit 11 (Week 19), and Visit 12 (Week 23).

#### Confirmatory factor analysis (CFA)

A CFA was performed using EQS version 6.1 to determine whether a total score was justified or whether multiple subscales were appropriate with the addition of the new items. CFAs were performed with the data from Visit 1 (Week 0) and from Visit 12 (Week 23). Model fit was assessed using Bentler’s comparative fit index (CFI), with a CFI ≥ 0.90 indicating an acceptable model fit. Additional parameters of model fit that were evaluated were the root mean square error of approximation (RMSEA) and weighted root mean square residual (WRMR).

#### Reliability

##### Internal consistency reliability

Internal consistency reliability (Cronbach’s α) addressed the extent to which individual items within an instrument were related to one another [16]. Cronbach’s α was calculated for the FSDS-DAO and FSDS-R at Visit 1, Visit 2, Visit 5, and Visit 12. There were no tests of statistical significance for these estimations; α > 0.70 were generally considered acceptable for group-level data [17].

##### Test–retest reliability

Test–retest reliability was examined using intra-class correlations (ICCs), Spearman’s correlations, and paired t-tests of FSDS-DAO and FSDS-R scores from Visit 1 to Visit 2. ICC values > 0.70 are generally considered acceptable for establishing test–retest reliability [18].

#### Validity

##### Convergent validity

To examine convergent validity, the pattern and magnitude of the relationships of the FSDS-DAO and FSDS-R total scores with the FSEP-R, FSFI subscales and total score, GAQ item scores, WITS-9 total score, and number of satisfying sexual events (SSEs) were examined at Visit 5 and Visit 12 using Spearman’s rank correlation coefficients. Convergent validity was supported by correlations > 0.40 with questionnaires measuring similar concepts. It was expected that these measures would be moderately correlated, indicating that they measured related constructs but that they would not be correlated over 0.80 (indicating that they measured the same construct). Those measures that were more directly related to sexual arousal and level of desire were expected to have higher correlations, while scales related to pain were expected to have lower correlations with the FSDS-DAO and FSDS-R scores and potentially demonstrate divergent validity.

##### Known-groups validity

The ability of the FSDS-DAO and the FSDS-R to differentiate among groups of participants according to known indicators such as treatment group or disease severity/clinical status at baseline (FSFI total score; FSFI arousal, desire, and satisfaction subscale scores; number of SSEs; and GAQ Items 1 and 2) was assessed using paired t-tests and general linear models (PROC GLM) with Scheffe’s post hoc comparisons to evaluate mean differences among participant subgroups at Visit 12.

#### Responsiveness

Several analytic approaches were taken to evaluate the responsiveness of the FSDS-DAO and FSDS-R. Changes in the total FSDS-DAO score were calculated from baseline (Visit 1; Week 0) to Visit 12 (Week 23) for the overall sample. Effect size [19] and responsiveness statistic were also calculated. Effect size was interpreted as small (0.20), moderate (0.50), or large (0.80) using Cohen’s convention [20]. The responsiveness statistic was computed by subtracting the placebo change score from the treatment change score and dividing by the standard deviation (SD) of the placebo change score ([treatment change score − placebo change score]/SD of placebo change score). The responsiveness statistic provided the magnitude of change between treatment groups.

### Ethical conduct

The study was conducted in accordance with Good Clinical Practice requirements, as described in guidelines of the International Conference on Harmonisation of Technical Requirements of Pharmaceuticals for Human Use (ICH) and in the Declaration of Helsinki. Each study site was reviewed by a central or local institutional review board (IRB) or ethics committee. The IRB approval numbers were Compass, 00519; WIRB, 20111036. Before any study procedures were initiated, written informed consent was obtained from each subject.

## Results

The sample used in these analyses consisted of all premenopausal women in the evaluable modified intent-to-treat (mITT) population of the phase 2b study who had FSDS-DAO scores at baseline and at ≥ 1 postrandomization follow-up visit. The mITT population was defined as all randomized subjects in the phase 2b study who took at least 1 outpatient dose of double-blind treatment (ie, 1 outpatient dose after the 2 in-clinic doses of double-blind treatment) and who had at least 1 follow-up visit. The number of study participants over time is shown in Table 1. The baseline characteristics for the safety population of the bremelanotide study are summarized in Table 2 and were similar across dose groups.

**Table 1:** Study schema^a^ and evaluable modified intent-to-treat population

	Visit 1 (screening, Week 0)	Visit 2 (end of no-drug qualification period, Week 4)	Visit 5 (end of single-blind placebo treatment period, Week 10)	Visit 12 (end of double-blind treatment period, Week 23)
Evaluable mITT	N = 325	N = 324	N = 238	N = 229
FSDS-DAO	X	X	X	X
FSFI	X	X	X	X
GAQ			X	X
WITS-9			X	X

FSDS-DAO = Female Sexual Distress Scale-Desire/Arousal/Orgasm; FSFI = Female Sexual Function Index; GAQ = General Assessment Questionnaire; mITT = modified intent-to-treat; WITS-9 = Women’s Inventory of Treatment Satisfaction

^a^The FSEP-R was completed after each sexual encounter

**Table 2:** Baseline characteristics (safety population)^a^

Characteristic	Placebo (N = 97)	Bremelanotide			All pooled (N = 394)
		0.75 mg (N = 100)	1.25 mg (N = 99)	1.75 mg (N = 98)	
Age (years), mean (SD)	37.0 (7.7)	37.6 (7.8)	35.7 (7.2)	37.0 (7.6)	36.9 (7.6)
*Race, n (%)*					
White	75 (77)	71 (71)	65 (66)	70 (71)	281 (71)
Black	19 (20)	25 (25)	32 (32)	23 (23)	99 (25)
Other	3 (3)	4 (4)	2 (2)	5 (5)	14 (3)
*Diagnosis, n (%)*					
FSAD	4 (4)	3 (3)	3 (3)	2 (2)	12 (3)
HSDD	24 (25)	20 (20)	24 (24)	24 (24)	92 (23)
Mixed	69 (71)	77 (77)	72 (73)	72 (73)	290 (74)
Menses frequency regular, n (%)	72 (74)	75 (75)	86 (87)	79 (81)	312 (79)
Used oral contraception ≤ 30 days before screening, n (%)	12 (12)	15 (15)	11 (11)	15 (15)	53 (13)

FSAD = female sexual arousal disorder; HSDD = hypoactive sexual desire disorder; SD = standard deviation

^a^All participants exposed to study drug

### FSDS-DAO psychometric evaluation

#### Instrument descriptive characteristics

The mean score for the FSDS-DAO items at Visit 1 (Week 0) ranged from 1.6 (Item 12, angry about your sex life) to 3.1 (Item 13, bothered by low sexual desire) and the mean total score was 39.4 ± 9.3. Scores were very similar at Visit 2 (Week 4, baseline), but improved (i.e., decreased) at Visit 5 (Week 10) with a mean total score of 32.2 ± 12.9 and at Visit 12 (Week 23) with a mean total score of 23.3 ± 14.6. A similar pattern of improvement was seen on the FSDS-R with a mean total score at baseline of 33.9.6 (± 8.3), 27.8 (± 11.4) at Visit 5, and 20.0 (± 12.7) at Visit 12.

For the FSDS-DAO, there were no floor effects (defined as > 25% of responses as “never”) observed at Visit 1 or Visit 2. Floor effects were observed for Item 12 (angry about your sex life) at Visit 5 and for 5 of the 15 items at Visit 12, including Item 6 (inferior because of sexual problems), Item 8 (sexually inadequate), Item 10 (embarrassed about sexual problems), Item 12 (angry about your sex life), and Item 15 (frustrated by problems with orgasm). No floor effects were observed on the FSDS-R except at Visit 12 (n = 22, 7.1%).

Ceiling effects (defined as > 25% of responses as “always”) were observed for Items 3 (guilty about sexual difficulties), 11 (dissatisfied with your sex life), and 13 (bothered by low sexual desire) at Visit 1, as well as Items 4 (frustrated by your sexual problems), 11, 13, and 15 (frustrated by problems with orgasm) at Visit 2 on the FSDS-DAO. There were no ceiling effects observed at Visits 5 or 12. On the FSDS-R, ceiling effects were observed for all time points: Visit 1 (n = 8, 2.5%), Visit 2 (n = 12, 3.8%), Visit 5 (n = 4, 1.2%), and Visit 12 (n = 5, 1.6%). There were no subjects with missing data on either analysis for either floor or ceiling effects.

#### Confirmatory factor analysis (CFA)

The FSDS-DAO items fit very well with a Bentler’s CFI [21] of 0.929 and WRMR of 1.704 (Table 3). Factor loadings at Week 1 ranged from 0.474 (frustrated by orgasm) to 0.813 (felt sexually inadequate). At Week 12, factor loadings ranged from 0.716 (frustrated by orgasm) to 0.935 (stressed about sex). The FSDS-R items fit well at both Visit 1 and Visit 12 with a CFI of 0.943 (Visit 1) and 0.985 (Visit 2) and a WRMR of 1.597 (Visit 1) and 1.451 (Visit 12). Factor loadings ranged from 0.613 (angry about your sex life) to 0.813 (sexually inadequate) for Visit 1, and 0.763 (angry about your sex life) to 0.937 (stressed about sex) for Visit 12.

**Table 3:** FSDS-DAO and FSDS-R item and total score analysis: CFA at visits 1 and 12

FSDS-DAO/FSDS-R item	Visit 1 factor loadings		Visit 12 factor loadings	
	FSDS-DAO	FSDS-R	FSDS-DAO	FSDS-R
1. Distressed about sex	0.766	0.775	0.883	0.886
2. Unhappy about sex relationship	0.759	0.798	0.922	0.924
3. Feel guilty regarding sexual difficulties	0.686	0.693	0.876	0.880
4. Frustrated by sexual problems	0.767	0.765	0.934	0.932
5. Stressed about sex	0.774	0.783	0.935	0.937
6. Inferior due to sexual problems	0.803	0.808	0.898	0.902
7. Worried about sex	0.785	0.781	0.909	0.912
8. Felt sexually inadequate	0.813	0.813	0.924	0.926
9. Regrets about sexual functioning	0.794	0.785	0.896	0.898
10. Embarrassed about sexual problems	0.679	0.683	0.870	0.875
11. Dissatisfied with sex life	0.737	0.725	0.908	0.907
12. Angry about sex life	0.606	0.613	0.760	0.763
13. Bothered by low desire	0.671	0.649	0.896	0.875
14. Concerned by arousal difficulties	0.636	N/A	0.877	N/A
15. Frustrated by orgasm	0.474	N/A	0.716	N/A
Confirmatory fit index	0.929	0.943	0.981	0.985
*RMSEA*				
Estimate	0.142	0.144	0.140	0.146
90% CI	0.132–0.152	0.132–0.156	0.130–0.151	0.134–0.158
WRMR	1.704	1.597	1.511	1.451

CFA = confirmatory factor analysis; CI = confidence interval; FSDS-DAO = Female Sexual Distress Scale-Desire/Arousal/Orgasm; N/A = not applicable; RMSEA = root mean square error of approximation; WRMR, = weighted root mean square residual

#### Reliability

##### Internal consistency reliability

The FSDS-DAO demonstrated adequate internal consistency reliability, with Cronbach’s α of 0.91, 0.94, 0.96, and 0.97 at Visits 1, 2, 5, and 12, respectively. Corresponding values for the FSDS-R were 0.91, 0.94, 0.96, and 0.97.

##### Test–retest reliability

Test–retest reliability of the FSDS-DAO was assessed using data from 318 participants at Visit 1 and Visit 2. The ICC was 0.61, indicating moderate stability [22]. Spearman’s correlation was 0.62 (*P* < 0.001), indicating acceptable test–retest reliability (Table 4). For the FSDS-R, the ICC was 0.63. Although not > 0.60, an ICC > 0.60 is considered to indicate substantial agreement [15].

**Table 4:** FSDS-DAO test–retest reliability

	N	Visit 1 Mean (SD)	Visit 2 Mean (SD)	T-value^a^	*P*-value	Spearman’s correlation	ICC
FSDS-DAO total score	318	39.3 (9.3)	40.1 (10.3)	− 1.75	0.0804	0.62^b^	0.61

FSDS-DAO = Female Sexual Distress Scale-Desire/Arousal/Orgasm; ICC = intra-class correlation coefficient; SD = standard deviation

^a^Paired t-tests comparing responses at Visit 1 and Visit 2

^b^*P* < 0.0001

#### Validity

##### Convergent validity

All correlations of the FSDS-DAO and the FSDS-R total scores with other PRO measures were statistically significant at Visits 5 (baseline) and 12 (end of double-blind period; Table 5 and Additional file 1: Table S1). For the FSDS-DAO, correlations were substantial with the FSFI total score and the FSFI desire, arousal, lubrication, orgasm, and satisfaction domain scores (ranging from r = − 0.41 to r = − 0.67); WITS-9 total score (r = − 0.55); and GAQ Items 1–3 at Visit 5 (ranging from r = − 0.41 to r = − 0.46). Similar and stronger relationships were observed at Visit 12. The strength of the correlation between the number of SSEs and the total FSDS-DAO declined between Visits 5 and 12 (r = − 0.42 and r = − 0.32, respectively). Divergent validity of the FSDS-DAO total score was supported by weak correlations at Visit 5 and Visit 12 with the FSFI Pain subscale score (r = − 0.17 and r = − 0.22, respectively), suggesting that sexual pain is a distinct measure of sexual distress as measured by the FSDS-DAO. All correlations were as expected in both their direction and magnitude (Table 5).

**Table 5:** FSDS-DAO versus specified PRO measures (evaluable mITT sample)

Comparator	Spearman’s rank correlation coefficient	
	End of baseline placebo self-dosing (N = 323)	End of double-blind study-drug use (N = 309)
FSFI total	− 0.65^b^	− 0.66^b^
FSFI desire	− 0.58^b^	− 0.63^b^
FSFI arousal	− 0.59^b^	− 0.64^b^
FSFI lubrication	− 0.44^b^	− 0.41^b^
FSFI orgasm	− 0.43^b^	− 0.55^b^
FSFI satisfaction	− 0.67^b^	− 0.66^b^
FSFI pain	− 0.17^c^	− 0.22^b^
GAQ Q1 (satisfaction with arousal)	− 0.46^b^	− 0.57^b^
GAQ Q2 (satisfaction with desire)	− 0.45^b^	− 0.56^b^
GAQ Q3 (benefit from study drug)	− 0.41^b^	− 0.54^b^
WITS-9 total	− 0.55^b^	− 0.64^b^
Number of SSEs per month^a^	− 0.42^b^	− 0.32^b^

FSDS-DAO = Female Sexual Distress Scale-Desire/Arousal/Orgasm; FSFI = Female Sexual Function Index; GAQ = General Assessment Questionnaire; mITT = modified intent-to-treat; PRO = patient-reported outcome; Q = Question; SSEs = satisfying sexual events; WITS = Women's Inventory of Treatment Satisfaction

^a^N = 314 at end of baseline and 267 at end of double-blind treatment

^b^*P* < 0.001

^c^*P* < 0.01

Convergent validity of the FSDS-R with other PRO questionnaires was demonstrated by correlations with items and total scores of the other scales (refer to Additional file 1: Table S1 for detailed data). As with the FSDS-DAO, weak correlations were observed for the FSFI orgasm, lubrication, and pain scores at various visits (Additional file 1: Table S1).

##### Known-groups validity

Known-groups validity was assessed by categorizing participants according to the number of SSEs and other PRO measures such as FSFI total and subscale scores and GAQ Items 1 and 2. The mean FSDS-DAO total score was significantly higher (worse) in women who reported < 2 SSEs (“yes” to FSEP-R Question 10) compared with women who reported ≥ 2 or more SSEs (29.4 vs 17.9; *P* < 0.001). Similarly, the mean FSDS-R total score was significantly higher in women who reported < 2 SSEs compared with women who reported at least 2 SSEs (25.3 vs. 15.4; *P* < 0.001).

**Fig. 1:**
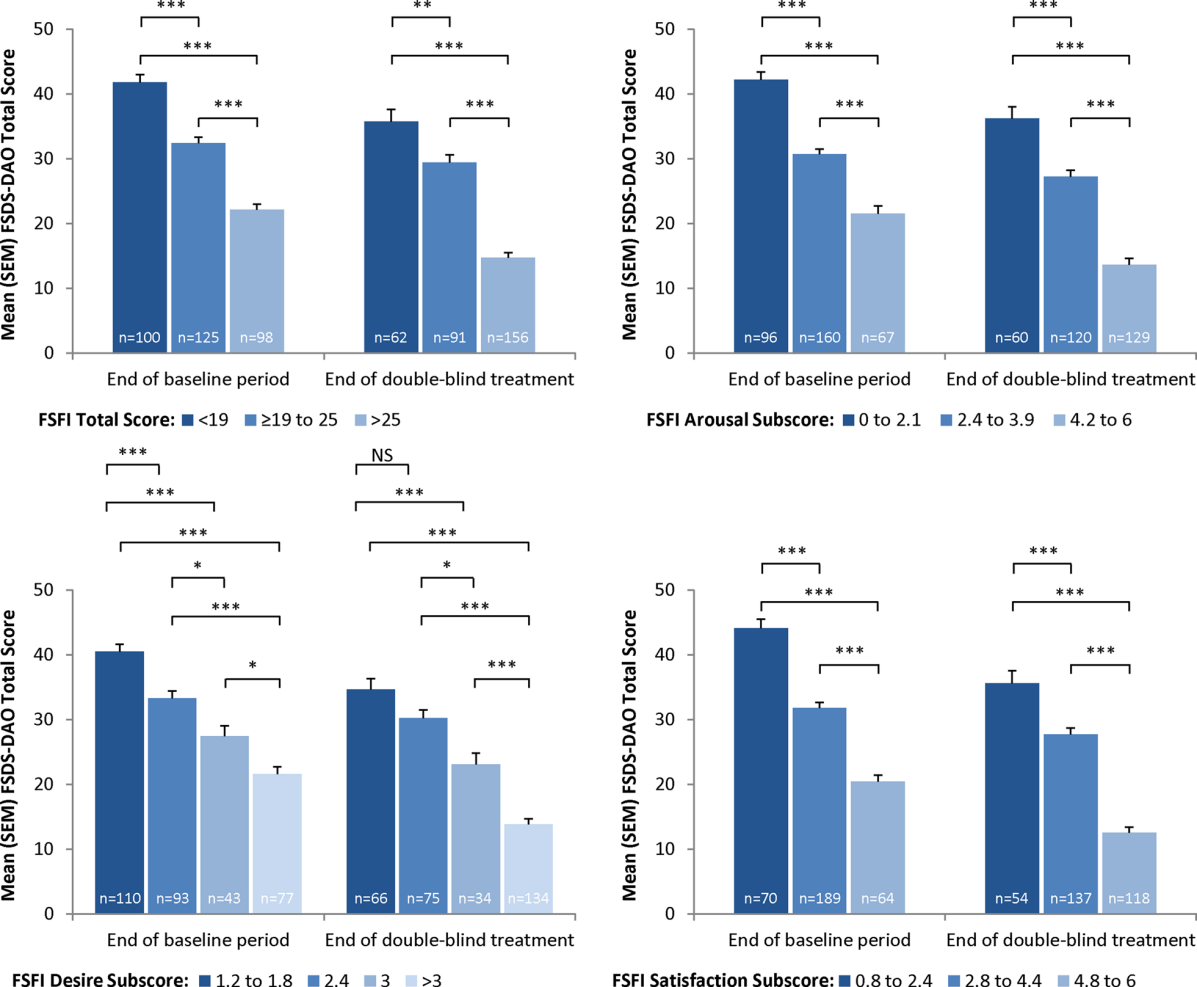
Discriminant validity: FSDS-DAO total score versus FSFI total and subscale scores (evaluable mITT sample). *P < 0.05; **P < 0.01; ***P < 0.001, Scheffe’s test. FSDS-DAO = Female Sexual Distress Scale-Desire/Arousal/Orgasm; FSFI = Female Sexual Function Index; mITT = modified intent-to-treat; NS = not significant; SEM = standard error of the mean

Known-groups validity was also assessed by examining the scores on the FSDS-DAO by distributional cut-points for the FSFI total score, desire subscale score, arousal subscale score, and satisfaction subscale score. As expected, for each of these comparisons at Visit 5 and Visit 12, those with the worst levels of sexual function on the FSFI also showed the worst FSDS-DAO scores, and a clear and consistent linear trend was observed of better FSDS-DAO scores for better levels of each FSFI domain. All but one of these comparisons were significant (all *P* < 0.05 except for FSFI Desire subscale score of 1.2 to 1.8 versus 2.4 at Visit 12; Fig. 1).

**Fig. 2:**
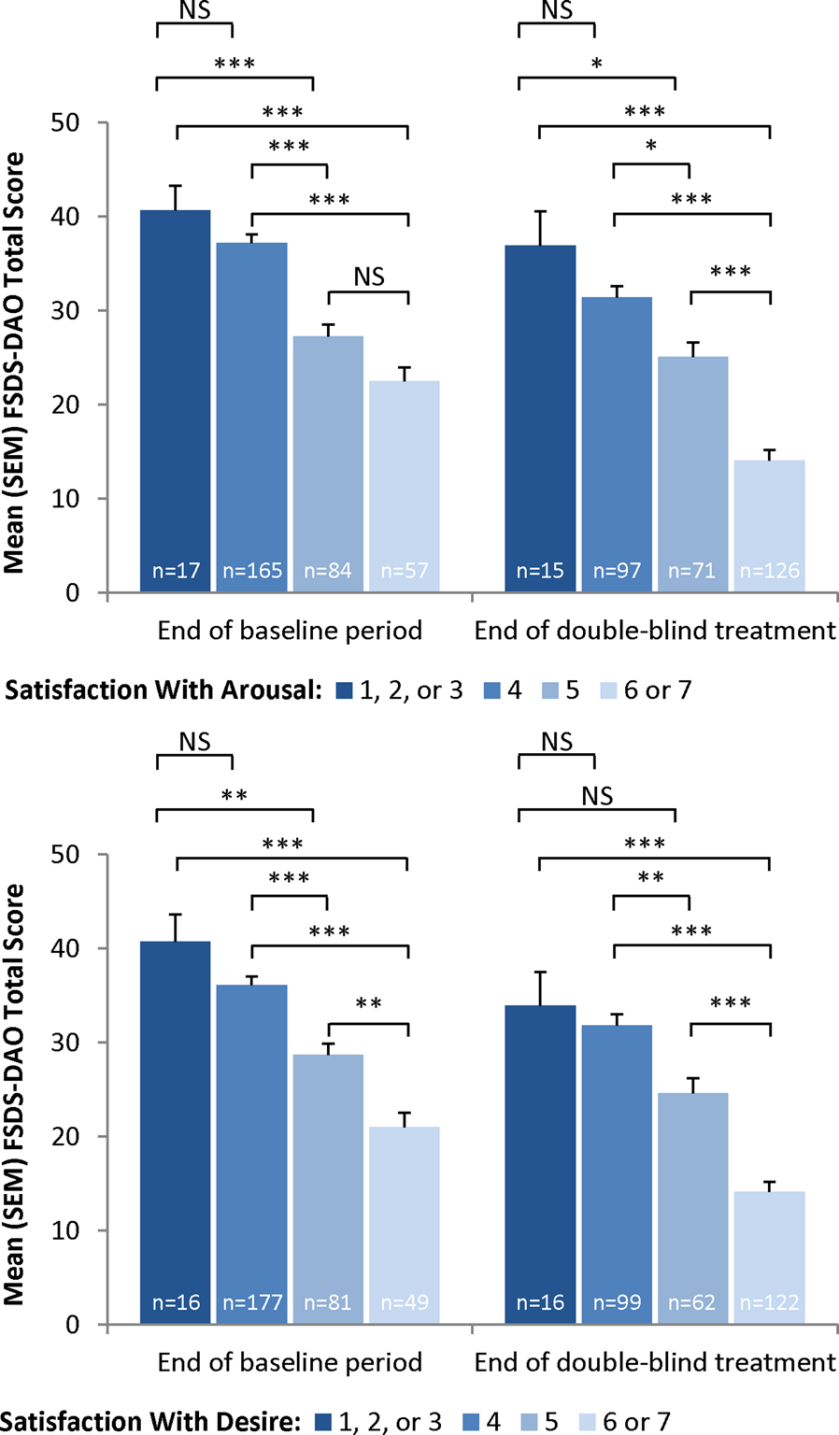
Discriminant validity: FSDS-DAO total score versus GAQ item scores (evaluable mITT sample). *P < 0.05; **P < 0.01; ***P < 0.001, Scheffe’s test. FSDS-DAO = Female Sexual Distress Scale-Desire/Arousal/Orgasm; GAQ = General Assessment Questionnaire; mITT = modified intent-to-treat; NS = not significant; SEM = standard error of the mean

Similar analyses were conducted by distributional cut-points for GAQ Items 1 (satisfaction with arousal) and 2 (satisfaction with desire). The FSDS-DAO discriminated at Visit 5 and Visit 12 between women who scored 1–3 and those who scored 5, between women who scored 1–3 and those who scored 6–7, between women who scored 4 and those who scored 5, and between women who scored 4 and those who scored 6–7 on both GAQ Items 1 and 2 (all *P* < 0.05 except Item 2 at Visit 12 for women who scored 1–3 and those who scored 5; Fig. 2). The FSDS-DAO was not able to discriminate at either visit on GAQ Items 1 and 2 between women who scored 1–3 and those who scored 4. In addition, the FSDS-DAO discriminated at Visit 5 for GAQ Item 2 and at Visit 12 on both items between women who scored 5 versus those who scored 6–7.

Known-groups validity was observed at Visit 12 between women who scored 1–3 and those who scored 5, between women who scored 1–3 and those who scored 6–7, between women who scored 4 and those who scored 5, and between women who scored 4 and those who scored 6–7 on GAQ item 1 (all *P* < 0.05). The FSDS-R was not able to discriminate at Visit 12 between women who scored 1–3 and those who scored 5 (*P* = 0.5078) for GAQ item 1. Known-groups validity was observed at Visit 12 between women who scored 1–3 and those who scored 6–7, between women who scored 4 and those who scored 5, and between women who scored 4 and those who scored 6–7 on GAQ item 2 (all *P* < 0.05). The FSDS-R was not able to discriminate at Visit 12 between women who scored 1–3 and those who scored 4 (*P* = 0.9384) nor between women who scored 1–3 and those who scored 5 (*P* = 0.0667) for GAQ item 2.

#### Responsiveness

Both the FSDS-DAO and FSDS-R scores changed over the course of treatment; the mean change score from Visit 1 to Visit 12 for the overall sample was –15.9 (*P* < 0.0001) on the FSDS-DAO and –13.9 (*P* < 0.0001) for the FSDS-R. Effect sizes were large at 1.71 and 1.65 (FSDS-DAO and FSDS-R, respectively), and the responsiveness statistic was 0.13 for both. The size of the responsiveness statistic was mainly driven by the change in the treatment not being much bigger than the change in the placebo group.

## Discussion

In these analyses, both the FSDS-DAO and FSDS-R demonstrated acceptable internal consistency reliability, test–retest reliability, construct validity, known-groups validity, and responsiveness, reliably assessing sexual-related distress in women with FSAD and/or HSDD. Our findings of internal consistency, test–retest reliability, and construct and known-groups validity for the FSDS-DAO demonstrate that the addition of the arousal and orgasm items did not impact the validity and reliability of the PRO measure. Acceptable construct validity, both convergent and divergent, was demonstrated by significant correlations with related PRO scales in the expected directions and magnitude. Test–retest reliability was acceptable between Visits 1 and 2 (4 weeks apart during the no-drug qualification period), with an ICC of 0.61 and a Spearman’s correlation coefficient score of 0.62. The FSDS-DAO demonstrated adequate internal consistency reliability, with Cronbach’s α ≥ 0.91 over the course of the clinical study. The CFAs for the FSDS-DAO and FSDS-R demonstrated some evidence supporting a single factor; however, the CFI (CFI = 0.93 and CFI = 0.94, respectively) and other model fit indices did not meet all recommended fit criteria, although the factor loadings ranged from 0.47 to 0.81 for the FSDS-DAO and 0.61 to 0.81 for the FSDS-R.

Previous psychometric evaluation of the FSDS-R in a sample of women with HSDD demonstrated better test–retest reliability for the correlation between Day 0 and Day 28 (ICC = 0.749) versus that observed for the FSDS-DAO in the current study (ICC = 0.61) [7]. Differences in test–retest reliability between these initial analyses may be attributed to the samples in each study, as the current study included patients with HSDD and/or FSAD, whereas the FDSD-R was evaluated in patients with HSDD only. Moreover, the Spearman’s correlation between the 2 scores of 0.62 was statistically significant (*P* < 0.001), indicating acceptable test–retest reliability. When the analyses were restricted to participants who had a change score on the FSFI total scores from Visit 1 to Visit 2 within 2 points, the Spearman’s correlation was 0.73 (*P* < 0.0001) and the ICC was 0.73 for the FSDS-DAO. It is important to note that when we reproduced our analysis using the FSDS-R, which lacks the arousal and orgasm items, the data were generally consistent with those observed with the FSDS-DAO. Thus, a total score is appropriate with or without the orgasm and arousal items.

During the study, SSE counts showed a decreasing correlation with FSDS-DAO total score, in contrast to the observation that subjects with the worst levels of sexual function as measured by the FSFI also had the worst FSDS-DAO scores. As an FSD measure, however, SSE counts have not been extensively validated. Indeed, the definition of HSDD, the most common FSD diagnosis [2], includes no criteria or constraints regarding the patient’s amount of sexual activity. Women with HSDD may frequently engage in sex without having an interest in it. They may do so out of a sense of obligation to their partner, to feel “normal,” or for a multitude of other reasons. Since the number of sexual events in which they participate may be determined by factors that have little to do with a patient’s own sexual interest, the association between event counts and HSDD measures on validated instruments may be, at best, modest.

The present analyses have several limitations that should be considered when interpreting the findings. First, the study utilized the FSDS-DAO to assess FSD-related distress solely in premenopausal women with a clinical diagnosis of HSDD and/or FSAD, and who by definition and eligibility criteria did not have female orgasmic disorder. Future research could be conducted including such individuals. Second, the study’s FSD analyses were based entirely on PROs. For FSD, however, patient-rated treatment effects and changes in symptoms are clearly the most relevant outcome measures—more so, given the paucity of clinical or biological FSD markers. Finally, the use of the FSDS-DAO total score as an inclusion criterion reduces the variability of the scale and limited correlation at baseline, but further measurement evaluation was also performed at later visits. Despite these limitations, the analyses provide strong evidence that the recently developed FSDS-DAO has demonstrated evidence supporting validity, reliability, and responsiveness. For clinical trials and other research, the FSDS-DAO is “fit for purpose” in offering a comprehensive assessment of the distress associated with FSD and may be used with the FSFI-desire domain score to cover the major components of an HSDD diagnosis: low desire and associated distress.

## Conclusions

The extensively evaluated FSDS-R is a well-characterized and reliable measure for assessing sexually related personal distress in women. Questions related to arousal and orgasm were added to the FSDS-R to permit an increase in the breadth of coverage for the instrument. These psychometric analyses show evidence of validity, reliability, acceptability, and responsiveness for the FSDS-DAO as a measure of sexually related personal distress in the HSDD/FSAD population. For clinical trials and other research, the FSDS-DAO is “fit for purpose” in offering a comprehensive assessment of the distress associated with FSD and may be paired with the FSFI-desire domain score to cover the key components of an HSDD diagnosis: low desire and associated distress.

## Supplementary Information

**Additional file 1: Table S1.** FSDS-R versus specified PRO measures (evaluable mITT sample).

## Data Availability

The dataset analyzed during the current study is available from the corresponding author on reasonable request.

## Abbreviations

CFA: Confirmatory factor analysis
CFI: Comparative fit index
CI: Confidence interval
FDA: US Food and Drug Administration
FSAD: Female sexual arousal disorder
FSD: Female sexual dysfunction
FSDS-DAO: Female Sexual Distress Scale-Desire/Arousal/Orgasm
FSDS-R: Female Sexual Distress Scale-Revised
FSEP-R: Female Sexual Encounter Profile-Revised
FSFI: Female Sexual Function Index
GAQ: General Assessment Questionnaire
GLM: General linear model
HSDD: Hypoactive sexual desire disorder
ICC: Intra-class correlation
ICH: International Conference on Harmonisation of Technical Requirements of Pharmaceuticals for Human Use
IRB: Institutional review board
mITT: Modified intent-to-treat
PRO: Patient-reported outcome
Q: Question
RMSEA: Root mean square error of approximation
SC: Subcutaneous
SD: Standard deviation
SEM: Standard error of the mean
SSE: Satisfying sexual event
STRAW: Stages of Reproductive Aging Workshop
WITS-9: Women’s Inventory of Treatment Satisfaction
WRMR: Weighted root mean square residual
