# Supplementary material for: Psychometric validation of the Female Sexual Distress Scale-Desire/Arousal/Orgasm
Source: J Patient Rep Outcomes. 2021 Sep 24;5:100. doi: 10.1186/s41687-021-00359-1 (PMC8463644; doi:10.1186/s41687-021-00359-1)
Supplement: Supplementary file 1 — Additional file 1: Table S1. FSDS-R versus specified PRO measures (evaluable mITT sample). [file 41687_2021_359_MOESM1_ESM.docx]

**SUPPLEMENTARY MATERIAL**

**Psychometric Validation of the Female Sexual Distress Scale–Desire/Arousal/Orgasm**

**eTable 1.** FSDS-R vs Specified PRO Measures (Evaluable mITT Sample)

| Comparator | Spearman’s rank correlation coefficient | |
| --- | --- | --- |
|  | End of baseline placebo self-dosing (Visit 5)  (N = 325) | End of double-blind study-drug use (Visit 12)  (N = 311) |
| FSFI total | –0.62^b^ | –0.65^b^ |
| FSFI desire | –0.57^b^ | –0.62^b^ |
| FSFI arousal | –0.56^b^ | –0.63^b^ |
| FSFI lubrication | –0.41^b^ | –0.39^b^ |
| FSFI orgasm | –0.38^b^ | –0.51^b^ |
| FSFI satisfaction | –0.66^b^ | –0.66^b^ |
| FSFI pain | –0.16^c^ | –0.22^d^ |
| GAQ Q1 (satisfaction with arousal) | –0.45^b^ | –0.55^b^ |
| GAQ Q2 (satisfaction with desire) | –0.44^b^ | –0.54^b^ |
| GAQ Q3 (benefit from study drug) | –0.40^b^ | –0.53^b^ |
| WITS-9 total | –0.55^b^ | –0.63^b^ |
| Number of SSEs per month^a^ | –0.41^b^ | –0.31^b^ |

^a^N = 316 at end of baseline and 269 at end of double-blind treatment.

^b^*P* < 0.0001.

^c^*P* = 0.0040.

^d^*P* = 0.0001

FSDS-R = Female Sexual Distress Scale–Revised; FSFI = Female Sexual Function Index; GAQ = General Assessment Questionnaire; mITT = modified intent-to-treat; PRO = patient-reported outcome; Q = Question; SSEs = satisfying sexual events; WITS-9 = Women's Inventory of Treatment Satisfaction.
